# The Lectin Pathway of Complement as a Sentinel for Nutritional and Metabolic Status: From Molecular Immunomodulation by Nutrients to Public Health Perspectives

**DOI:** 10.3390/nu18162635

**Published:** 2026-08-12

**Authors:** Tomasz Olszowski, Dariusz Chlubek

**Affiliations:** 1 Department of Public Health, Epidemiology and Behavioral Genetics, Pomeranian Medical University in Szczecin, 70-111 Szczecin, Poland; tomasz.olszowski@pum.edu.pl; 2 Department of Biochemistry and Medical Chemistry, Pomeranian Medical University in Szczecin, 70-111 Szczecin, Poland

**Keywords:** lectin pathway, divalent cations, zinc inhibition, fat-soluble vitamins, nuclear receptors, gestational diabetes mellitus, somatotropic axis, microvascular complications, nanoplasmonic detection

## Abstract

The lectin pathway (LP) of complement activation functions as a crucial effector of innate immunity and a homeostatic sensor operating at the intersection of systemic metabolism, nutritional status, and endothelial integrity. This review provides a comprehensive synthesis of current molecular, clinical, and epidemiological literature regarding the environmental and metabolic regulation of this pathway. First, we summarize the biophysical, structural, and stoichiometric requirements for divalent cations in fluid-phase activation and macromolecular assembly, integrating the contrasting roles of calcium (Ca^2+^) and zinc (Zn^2+^) into an explanatory Dual-Cation Dichotomy Framework. Second, we evaluate the transcriptomic mechanisms of nutrigenetic licensing, reviewing how fat-soluble vitamins sustain endoplasmic reticulum chaperone networks and mucosal barrier competence. We discuss how these micronutrient-driven axes interact with host genetic diversity, presenting a Nutrigenetic Rescue Framework to contextualize the environmental modulation of low-expressing *MBL2* alleles. Third, the LP responds to metabolic and endocrine shifts, focusing on its biomarker value in gestational diabetes and its clinical patterns in type 1 diabetes. These connections to metabolic disease and related microvascular complications are further integrated into a broader Somatotropic-Gestational Sentinel Hypothesis. Fourth, we connect these metabolic profiles with public health challenges, reviewing LP hyperactivation in viral infections and discussing localized surface plasmon resonance (LSPR) biosensors, at present a conceptual, preclinical technology, as a candidate approach for future point-of-care population screening. In conclusion, bridging nutritional biochemistry with metabolic endocrinology and diagnostic technologies that remain largely preclinical outlines a potential shift from empiric management toward biomarker-driven, point-of-care stratification, which could help mitigate both infectious thromboinflammation and chronic microvascular failure once these technologies undergo further mechanistic and clinical validation.

## 1. Introduction

Historically, the lectin pathway (LP) of complement activation has been characterized predominantly through an immunological framework as an autonomous cascade driving innate host defense against microbial pathogens. However, current clinical biochemistry and molecular epidemiology indicate that the LP also functions as a homeostatic sensor operating at the intersection of systemic metabolism, nutritional status, and endothelial integrity [1,2,3]. Rather than functioning via a binary activation mode, the pathway relies on a molecular architecture driven by complex macromolecular multimers. The system comprises a coordinated assembly of soluble pattern recognition molecules (PRMs), including mannose-binding lectin (MBL), ficolin-1, ficolin-2, ficolin-3, and collectin-11 (CL-11). These recognition molecules are physically interlinked with resident lectin pathway-associated serine proteases, namely MASP-1, MASP-2, MASP-3, and their structural non-enzymatic regulatory proteins, MAp44 and MAp19 [1,2] (Table 1). Within this framework, circulating co-complexes of MASP-1 and MASP-2 associated with a single PRM scaffold drive cascade activation and downstream signaling in a competitive manner regulated by the endogenous inhibitory capacity of MAp44 [3,4].

The baseline configurations, circulating complex dynamics, and kinetic activation thresholds of these components establish a distinct physiological equilibrium in human serum. High-resolution mapping of absolute concentration profiles in healthy cohorts alongside the identification of unique macromolecular hybrid formations, such as circulating cross-pathway C1q/MASP complexes, confirm that the system maintains a calibrated biochemical steady-state designed to recognize biochemical deviations in blood chemistry [5,6]. Because these macromolecular complexes require specific conformational configurations and stable metabolic homeostasis to maintain this baseline balance, the pathway acts as a molecular sensor capable of translating systemic microenvironmental shifts into downstream endothelial and metabolic responses [2,3]. This baseline capacity is integrated into host physiology; the hepatocyte synthesis and secretion of core pattern-recognition components are directly subject to active endocrine regulation, insulin dynamics, and systemic metabolic signaling, linking the pathway’s functional availability to the host’s overall metabolic status [7].

**Figure 1:**
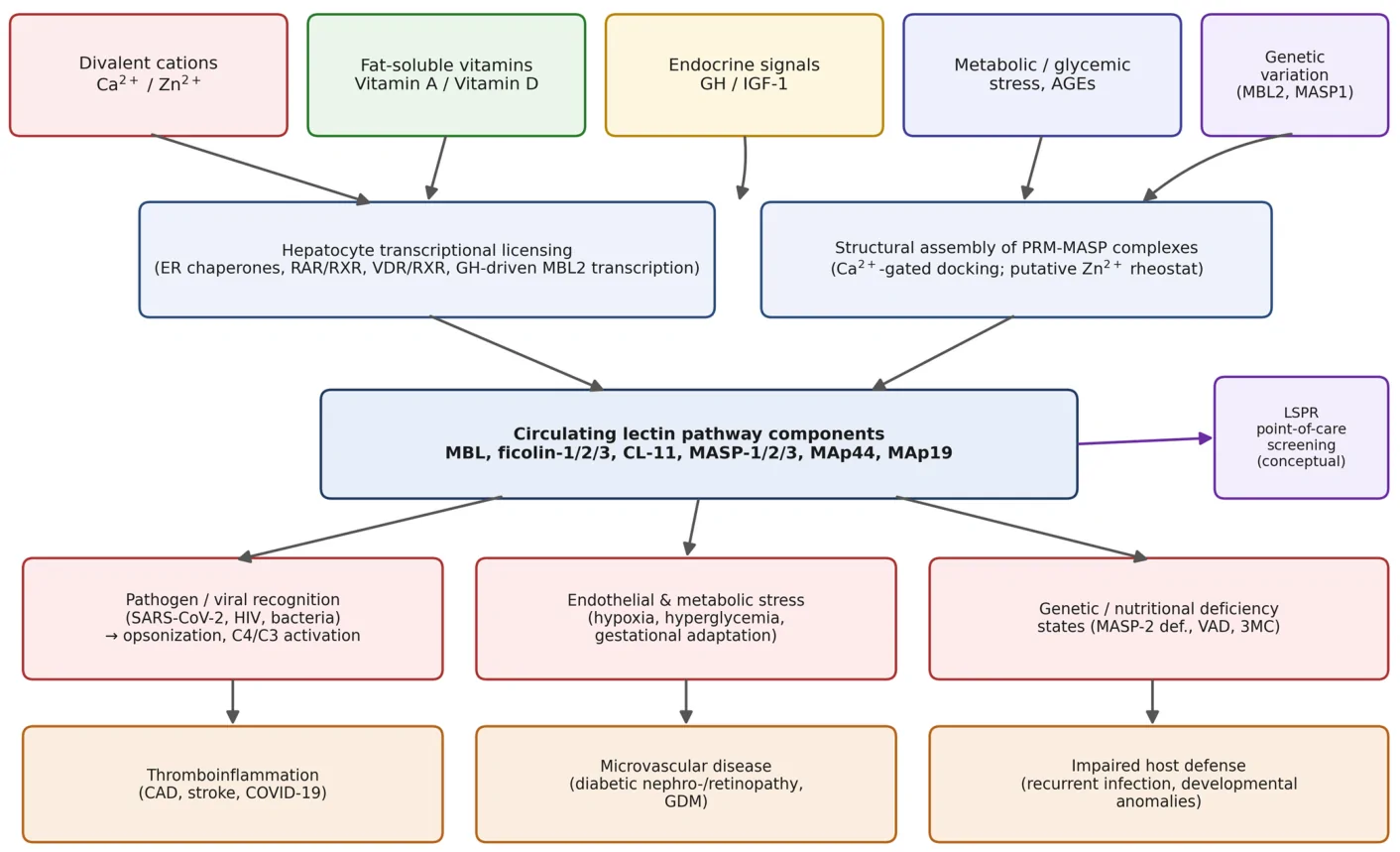
Convergent regulation of the complement lectin pathway (LP) by nutritional, endocrine, genetic and metabolic inputs, and its downstream clinical and diagnostic implications. Abbreviations: 3MC: 3MC syndrome; AGEs: Advanced glycation end-products; C3: Complement component 3; C4: Complement component 4; CAD: Coronary artery disease; Ca^2+^: Calcium ion; CL-11: Collectin-11; COVID-19: Coronavirus disease 2019; ER: Endoplasmic reticulum; GDM: Gestational diabetes mellitus; GH: Growth hormone; HIV: Human immunodeficiency virus; IGF-1: Insulin-like growth factor 1; LSPR: Localized surface plasmon resonance; MAp19: 19 kDa MBL-associated protein; MAp44: 44 kDa MBL-associated protein; MASP-1/2/3: MBL-associated serine protease (-1/-2/-3); MBL: Mannose-binding lectin; *MBL2*: Mannose-binding lectin 2 gene; PRM: Pattern recognition molecule; RAR: Retinoic acid receptor; RXR: Retinoid X receptor; SARS-CoV-2: Severe acute respiratory syndrome coronavirus 2; VAD: Vitamin A deficiency; VDR: Vitamin D receptor; Zn^2+^: Zinc ion.

Consequently, the functional competence and activation thresholds of these innate sensors are modulated and transcriptionally licensed by circulating micronutrients, genetic variation, and metabolic drivers, establishing the LP as a continuous monitor of the host’s metabolic status [8,9,10] (Figure 1). This systemic surveillance function represents a true nutrigenetic axis, where specific genetic variations in host innate sensors interact with micronutrient availability, such as vitamin A status or vitamin D target gene transcriptional receptor pathways. These gene–nutrient interactions dictate downstream inflammatory outcomes and mucosal barrier security [8,10,11]. When chronic metabolic stress, severe infectious challenges, or inherited genetic disruptions, such as human functional MASP-2 deficiency caused by structural missense mutations, alter this macromolecular stoichiometry, the homeostatic equilibrium of the pathway is compromised. Under these pathological conditions, unregulated or uncomplexed serine proteases initiate aberrant, cross-system proteolytic cascades at the endothelial interface [3,12,13]. This enzymatic dysregulation induces a prothrombotic endothelial phenotype and accelerates microvascular injury, thereby providing a direct biochemical link between acute innate immune hyperactivation, microvascular angiopathies, and systemic metabolic comorbidities.

Literature search strategy: this is a narrative, rather than systematic, review. PubMed/MEDLINE and Scopus were searched from January 2000 to June 2026 using combinations of the terms “lectin pathway,” “mannose-binding lectin,” “MASP,” “ficolin,” “calcium,” “zinc,” “vitamin A,” “vitamin D,” “gestational diabetes,” “type 1/2 diabetes,” “complement,” and “surface plasmon resonance.” One foundational study predating this window, on serine-protease–zinc coordination chemistry, was additionally identified through citation tracking of the retrieved literature and included because it remains the primary source for a specific mechanistic claim discussed in Section 2.4. Original research in humans and validated animal models was prioritized over in vitro-only or purely computational studies when both were available for a given claim; non-English-language and non-peer-reviewed sources were excluded. Because this is a narrative synthesis rather than a systematic review with predefined inclusion/exclusion criteria, selection bias cannot be excluded, and the explanatory frameworks proposed in Section 2, Section 3, Section 4 and Section 5 (summarized in Table 2) should be read as an interpretive synthesis rather than a formally graded evidence base.

## 2. Divalent Cations as Molecular Regulators: The Dichotomy of Calcium and Zinc in Lectin Pathway Activation

The Introduction outlined the lectin pathway as a homeostatic sensor whose competence depends on nutritional and metabolic inputs. Before this sensing function can be examined at the transcriptional, endocrine, or clinical level, however, its most immediate physical requirement must be established: the divalent cations required to assemble the pattern-recognition complexes in the first place. The following section therefore begins at this most fundamental level of regulation.

### 2.1. Biophysical and Stoichiometric Requirements for Ca^2+^ in Macromolecular Assembly

The structural integrity, stoichiometric composition, and fluid-phase activation of the complement lectin pathway (LP) are regulated by microenvironmental calcium (Ca^2+^) gradients. Utilizing surface plasmon resonance (SPR) and gel filtration analyses, Thielens et al. [1] demonstrated that human MBL-associated serine proteases (MASP-1 and MASP-2), alongside the non-catalytic splice variant MAp19, circulate in human serum as stable homodimers. These homodimeric configurations are mediated by their *N*-terminal CUB1-EGF-CUB2 segments and exhibit distinct sedimentation coefficients ranging from 2.8 S to 3.2 S strictly in the presence of physiological calcium concentrations. Importantly, surface plasmon resonance (SPR) data revealed an absence of direct inter-protease hybridization in solution, indicating that human serum contains distinct populations of MBL-MASP-1 and MBL-MASP-2 complexes rather than mixed-protease multimers. While the dimeric interface of MASP-1 is structurally insensitive to chelation, its physical docking onto the collagenous stalk of MBL requires Ca^2+^ coordination within a conserved calcium-binding pocket located inside the EGF domain. This domain functions as a conformational switch. This interaction follows a strict thermodynamic hierarchy: full-length MASP-2 and MASP-1 bind MBL with high affinity, with dissociation constants (KD) of 0.8 nM and 1.4 nM, respectively.

In vivo, this calcium-dependent macro-assembly is validated by the serum stoichiometric profiles established by Thiel et al. [2]. Under normocalcemic conditions, circulating MASP-1 fractionates within large, stable pattern-recognition complexes eluting at approximately 600 kDa during size-exclusion chromatography. Shifting to a chelating EDTA environment, however, completely dismantles these macromolecular structures into individual 75 kDa monomeric chains, demonstrating the structural necessity of calcium for complex stability. On an epidemiological scale, multiplex immunoassays demonstrated that MASP-1 is highly abundant in the adult circulation, maintaining a log-normal distribution with a median concentration of 11 µg/mL. Importantly, despite sharing a near-identical *N*-terminal structural framework with its sister splice variants MASP-3 and MAp44, no statistically significant correlation governs the circulating levels of these three *MASP1* gene products (*p* > 0.05). This expression autonomy implies that competition for a limited set of calcium-licensed positions on the MBL collagenous stalk is driven dynamically by the localized thermodynamic availability of each distinct homodimer rather than being coordinately programmed at the transcriptional level.

The fluid-phase architecture is dynamic and polydisperse compared to the rigid, pre-assembled C1q-C1r2-C1s2 complex of the classical pathway. As elucidated at the macromolecular level by Degn et al. [4], although MASP-1 and MASP-2 do not form heterodimers in isolation, the engagement of Ca^2+^-licensed positions on a single carbohydrate-binding stalk forces the assembly of functional MASP-1–MASP-2 co-complexes. This spatial juxtaposition is biologically mandatory: functional assays utilizing recombinant proteins proved that MASP-1 must physically transactivate adjacent MASP-2 zymogens to initiate the sequential cleavage of C4 and C2, which is required for canonical C4b-C2a convertase assembly. Furthermore, Degn et al. demonstrated that this activation framework is endogenously modulated by MAp44, which impairs complement progression not merely through competitive displacement of MASP-2, but by disrupting these functional co-complexes and preventing the necessary transactivation event [4].

This calcium gating extends beyond canonical LP boundaries to modulate heterotypic cross-talk between distinct complement axes. Utilizing microscale thermophoresis (MST) and immunoprecipitation assays, Rosbjerg et al. [6] documented the physiological existence of circulating hybrid complexes merging classical and lectin pathway components. Their data established that the classical pattern-recognition molecule, C1q, acts as an in vivo docking platform for lectin pathway proteases, forming functional C1q-MASP-2 and C1q-MASP-3 co-complexes in human serum. These interactions display high-nanomolar affinities and strict calcium dependence, undergoing prompt dissociation into individual subcomponents upon chelation by EDTA. Structural competition assays confirmed that zymogen MASPs utilize identical molecular interfaces (CUB1-EGF-CUB2) to engage C1q as the classical C1r_2_C1s_2_ tetramer, proving that the physical assembly of these heterotypic protease platforms is driven entirely by Ca^2+^-licensed interactions.

These matrix-level dynamics are directly reflected in clinical screening protocols. Troldborg et al. [5] demonstrated that the choice of anticoagulant for blood sampling alters the quantifiable levels of almost all lectin pathway components within a healthy reference cohort. Significantly lower serum levels of MASP-2 and MAp44 indicate their selective consumption during calcium-dependent clot formation. In contrast, the markedly higher concentration of M-ficolin observed in EDTA plasma stems from its ex vivo, chelation-induced release from intracellular granule stores within circulating granulocytes and monocytes during sample processing.

Collectively, these findings show calcium acting as a structural prerequisite for assembling and stabilizing the PRM-MASP complexes that initiate lectin pathway activation. As the next section shows, this dependence begins even earlier, inside the hepatocyte secretory pathway.

### 2.2. Intracellular Quality Control and Cationic Homeostasis in the Endoplasmic Reticulum

The dependency of LP components on the microenvironmental cation balance is initiated during biosynthesis within the endoplasmic reticulum (ER) lumen of hepatocytes, where expression levels are modulated by exogenous toxicogenomic factors. Toxicogenomic profiling and in silico toxicology models by Baralić et al. [24] identified the *MBL2* gene as a primary hub node within the transcriptional interactome affected by human exposure to per- and polyfluoroalkyl substances (PFAS). Specifically, their quantitative models demonstrated that long-chain legacy PFAS, including perfluorodecanoic acid (PFDA) and perfluoroundecanoic acid (PFUnDA), significantly downregulate *MBL2* mRNA expression, linking these xenobiotics directly to pathways associated with functional mannose-binding lectin deficiency. Furthermore, exposure to these PFAS mixtures disrupts hepatic lipid homeostasis via the alteration of *PPAR-α* signaling and fatty acid metabolism, while simultaneously accelerating intracellular oxidative stress and lipid peroxidation.

Physiologically, nascent intracellular MBL within the ER lumen functions as a co-chaperone required to maintain cellular homeostasis. As demonstrated by Chen et al. [25], MBL physically stabilizes the complex between the ER-resident chaperone BiP (immunoglobulin heavy-chain binding protein) and the transmembrane stress sensor PERK (protein kinase RNA-like endoplasmic reticulum kinase). Under conditions of MBL deficiency or transcriptional repression, the accelerated dissociation of the BiP-PERK interactome triggers the downstream PERK-ATF4-CHOP signaling cascade. This unfolded protein response upregulates the expression of *ERO1a* (endoplasmic reticulum oxidoreductase 1 alpha), driving subsequent inositol 1,4,5-trisphosphate receptor (IP_3_R)-mediated calcium efflux from the ER lumen into the cytosol, culminating in hepatic metabolic failure and apoptosis.

Importantly, the relationship between systemic zinc status and these hepatic components in early life appears independent of baseline expression. In a cross-sectional study of 398 neonates from the Danish Newborn Screening Biobank, Kyvsgaard et al. [26] quantified neonatal whole-blood zinc (WB-zinc) using laser ablation inductively coupled plasma mass spectrometry (LA-ICP-MS) and measured corresponding MBL concentrations via a multiplexed flowmetric immunoassay. The data revealed no statistically significant association between systemic WB-zinc content and circulating MBL levels, a finding that remained consistent when stratified by future autoimmune risk status. This indicates that baseline zinc status does not influence the hepatic synthesis or steady-state plasma concentration of MBL. This suggests that any regulatory footprint of zinc must operate downstream through direct structural or kinetic modulation of the activated protease complexes rather than altering baseline protein abundance.

MBL biosynthesis is itself cation- and toxicant-sensitive, coupling hepatocyte ER stress responses to lectin pathway output before secretion ever occurs. The next section considers what these secreted complexes do once they reach the vascular endothelium.

### 2.3. Endothelial Activation, Angiogenesis, and Membrane Calcium Kinetics

Downstream of hepatic secretion, functional MBL-MASP complexes interact directly with cellular membranes to modulate both intracellular calcium oscillations and vascular endothelial endpoints. At the epithelial interface, MBL serves as a non-canonical restrictor of store-operated calcium entry (SOCE) across the plasma membrane, thereby suppressing the signaling cascade driving epithelial–mesenchymal transition (EMT). Mechanism-focused insights by Liu et al. [27] established that upon internalization, MBL directly interacts with the cytosolic kinase 3-phosphoinositide-dependent kinase-1 (PDK1), promoting its ubiquitination and subsequent proteasomal degradation. Under physiological conditions, this MBL-dependent suppression of PDK1 attenuates downstream serum/glucocorticoid-regulated kinase 1 (SGK1) activity, which destabilizes *Orai1*, the pore-forming channel subunit of the SOCE machinery. Conversely, MBL deficiency prevents PDK1 ubiquitination, accelerating downstream SGK1 signaling and structural stabilization of *Orai1*, which culminates in persistent intracellular calcium elevation and pro-metastatic cellular remodeling.

Within the vascular compartment, the activation and substrate specificity of the mannan-binding lectin-associated serine proteases operate as cooperative proteolytic networks that bridge innate immunity and thrombosis [28]. Mechanistically, MASP-1 acts as the primary initiator of the complement response, whose catalytic activity is required to cross-activate MASP-2 and trigger downstream system conversion, while simultaneously interacting with non-complement proteolytic systems such as the coagulation machinery. Németh et al. [29] demonstrated that complement MASP-1 acts in synergy with other proinflammatory drivers, such as histamine and lipopolysaccharide (LPS), to enhance endothelial cell activation. Functionally, their work showed that MASP-1 triggers rapid endothelial calcium (Ca^2+^) mobilization via the cleavage of protease-activated receptors (PARs), which cooperatively upregulates the expression of cell adhesion molecules (E-selectin, VCAM-1, and ICAM-1) and exacerbates localized inflammatory feedback loops.

The physiological relevance of this MASP-1-mediated endothelial modulation is further emphasized during tissue repair, establishing a direct link to angiogenic endpoints. Németh et al. [30] revealed that complement MASP-1 modifies endothelial wound healing and angiogenesis by altering the transcriptomic profile of primary human umbilical vein endothelial cells (HUVECs) and accelerating CREB (cAMP response element-binding protein) phosphorylation. Notably, their functional assays showed that while individual treatments of mechanical wounding and MASP-1 both induce independent Ca^2+^ mobilization. However, the prior execution of mechanical wounding actively inhibits subsequent MASP-1-induced Ca^2+^ flux, illustrating a temporal desensitization of the endothelial calcium machinery during active cellular migration.

Beyond opsonization, MBL and MASP-1 also directly shape endothelial calcium signaling, barrier integrity, and wound-healing responses, independently of pathogen recognition. These calcium-dependent effects motivate the unifying framework of cation regulation proposed next.

### 2.4. The Dual-Cation Dichotomy Framework: Ca^2+^ Architectural Gating Versus Zn^2+^ Metalloprotease Rheostat

It should be emphasized at the outset that, unlike the calcium-dependence data reviewed in Section 2.1, Section 2.2 and Section 2.3, no lectin-pathway-specific biochemical study has yet directly demonstrated an inhibitory effect of zinc on MASPs; the zinc arm of the framework proposed below is a testable hypothesis extrapolated from general zinc–serine-protease coordination chemistry [14], not an established mechanism. Integrating these biophysical, intracellular, and endothelial findings, we propose an explanatory framework governed by a dual-cation dichotomy, wherein Ca^2+^ and Zn^2+^ act as distinct, asymmetrical regulators of the lectin pathway. Within this conceptual framework, structural calcium (Ca^2+^) operates as an architectural switch required for the pattern-recognition phase, the physical integrity of the PRM-MASP complexes, and the cross-talk stability of heterotypic C1q/MASP platforms. Conversely, we hypothesize that microenvironmental zinc (Zn^2+^) fluctuations function as a localized catalytic and homeostatic rheostat that directly modulates downstream enzymatic velocity and cellular resilience, a regulatory dimension that remained unexamined in the original cross-sectional and structural studies.

At the macromolecular level, while the initial docking of MASPs onto PRMs relies on Ca^2+^-dependent coordination spheres within the EGF domain, the subsequent kinetics of intermolecular transactivation, and the vulnerability of the complex to MAp44-mediated displacement, are hypothesized to be modulated by local zinc levels. Zinc availability may dictate the conformational flexibility of the serine protease (SP) domains, potentially controlling the transition from a dormant proenzyme co-complex to an active, cleaving protease network. Pathological Zn^2+^ shifts could induce alternative conformational constraints, altering zymogen binding orientations and perturbing the spatial alignment required for MASP-1 autoactivation, thereby either triggering premature proteolysis under metabolic stress or arresting canonical downstream propagation.

This dual-cation equilibrium is equally relevant within the intracellular compartment and at the plasma membrane. While standard Ca^2+^ coordination within the carbohydrate-recognition domains (CRDs) of MBL is mandatory to maintain its tertiary conformation and co-chaperone capacity (stabilizing PDK1 at the membrane and the BiP-PERK complex in the ER to prevent *Orai1*-mediated Ca^2+^ influx), xenobiotic-induced or oxidative stress can mobilize intracellular free Zn^2+^ pools. We hypothesize that an altered Zn^2+^/Ca^2+^ ratio favors competitive zinc binding within the evolutionarily conserved coordination spheres of MBL. This cation switch is expected to induce aberrant conformational constraints, destabilizing the MBL-BiP-PERK complex and triggering the IP_3_R-mediated calcium depletion cycle.

Finally, this microenvironmental Zn^2+^/Ca^2+^ ratio could dictate vascular endpoints; by competitively substituting for Ca^2+^ within the PRM-MASP architecture, pathological zinc accumulation may alter the catalytic domain visibility of MASP-1. Such a mechanism could disrupt its temporal cross-desensitization with mechanical wounding, potentially suppressing canonical sequential complement activation while amplifying PAR-dependent endothelial inflammatory loops.

Section 2 and Section 2.4 established that the lectin pathway’s functional competence begins with cation-dependent structural assembly, itself gated by calcium availability. That structural foundation, however, only explains whether the pathway’s protein components can assemble correctly—not whether enough of them are transcribed and secreted in the first place. The present section therefore turns to a second, largely independent layer of environmental control: how fat-soluble vitamin signaling, acting through nuclear hormone receptors, licenses the transcriptional machinery and endoplasmic reticulum chaperone capacity that determine baseline pattern-recognition molecule output.

In summary, calcium’s architectural role in the lectin pathway is well established, whereas zinc’s proposed regulatory role remains a hypothesis awaiting direct biochemical testing.

## 3. Nutrigenetic Licensing: The Vitamin A-D Axis and Innate Immune Sensor Competence

### 3.1. Nuclear Receptor Signaling and Transcriptional Regulation of ER Chaperone Networks

The transcriptional and functional competence of the lectin pathway (LP) is modulated by micronutrient-driven nuclear receptor signaling pathways that regulate cellular homeostatic networks within the endoplasmic reticulum (ER). On a molecular level, all-trans retinoic acid (ATRA) functions as an upstream regulator of ER quality control mechanisms. Characterizing downstream cellular adaptation, evidence regarding all-trans retinoic acid (ATRA) exposure demonstrates its capacity to modulate stress-responsive signaling networks and cellular differentiation profiles [31]. Signaling through retinoic acid receptors alters the baseline functional environment, interacting with pathways that manage proteotoxic strain and sustain endoplasmic reticulum competence, which indirectly supports the cellular machinery required for pattern-recognition molecule synthesis [31].

Conversely, the disruption of this homeostatic framework under states of nutritional deprivation induces systemic immune and barrier vulnerability. Transcriptomic profiling and total RNA sequencing executed by Chai et al. [8] under dietary vitamin A deficiency (VAD) revealed a pronounced, tissue-specific downregulation of gene networks governing mucosal barrier integrity and innate antimicrobial defenses, presenting distinct profiles in the small intestine versus the colon. Specifically, in vivo VAD models exhibited a marked reduction in transcripts encoding key tight junction proteins (such as claudin-1 and occludin). Crucially, co-expression module analyses in the small intestine identified that VAD directly suppresses the transcription of mannose-binding lectin 2 (*MBL2*) and ficolin. This demonstrates that physiological retinoid signaling via retinoic acid receptors (RARs) and retinoid X receptors (RXRs) is fundamentally required to sustain the baseline expression of core lectin pathway pattern-recognition components at the mucosal interface.

This retinoid-mediated transcriptional control operates in tandem with the vitamin D receptor (VDR) signaling pathway to establish full host sensor competence, particularly during early development. As reviewed by Cunningham-Rundles et al. [11] in the context of neonatal immune system maturation, active 1,25-dihydroxyvitamin D3 [1,25(OH)_2_D3] acts as a high-affinity ligand for the nuclear receptor VDR, which upon activation heterodimerizes with RXR. This heterodimeric complex binds directly to conserved vitamin D response elements (VDREs) located within the promoter regions of target genes regulating cellular differentiation, mucosal barrier integrity, and innate immune responses. Cunningham-Rundles et al. emphasized that single nucleotide polymorphisms (SNPs) and structural expression variations in the human *VDR* gene dictate host resistance, modulate downstream toll-like receptor (TLR) signaling, and govern inflammatory pathways. This underscores the requirement for coordinated fat-soluble vitamin axes in sustaining baseline defense mechanisms from the earliest stages of life.

Retinoid and vitamin D receptor signaling jointly license the transcriptional machinery required to sustain baseline pattern-recognition molecule expression and mucosal barrier competence, a capacity that depends, as discussed next, on additional antioxidant and epigenetic safeguards.

### 3.2. Transcriptomic Mapping, Antioxidant Microenvironments, and Epigenetic Barriers

The molecular integration within this fat-soluble vitamin axis has been further characterized by in vivo transcriptomic mapping of the vitamin D receptor (VDR) transcriptional network. Utilizing longitudinal total RNA sequencing (RNA-seq) and network biology models from human peripheral blood mononuclear cells, Jarosławska et al. [10] established that systemic vitamin D signaling comprehensively modulates core gene networks intersecting with innate pattern recognition and antigen-presentation pathways. Their mapping confirmed that activated VDR immunomodulates homeostatic processes, including autophagy and vacuole assembly, ensuring baseline transcriptional readiness of the innate immune response. This systemic protective blueprint is further extended to organ-specific endothelial and epithelial barriers; as reviewed by Liao et al. [32], adequate vitamin D signaling exerts important counter-regulatory oversight over pathological transmembrane signaling. It specifically suppresses the suPAR–β3 integrin axis, thereby mitigating downstream inflammatory cascade activation and preserving microvascular barrier integrity during acute immunometabolic stress.

Consequently, this transcriptional and receptor-level oversight operates under structural constraints where vitamin D status dictates cellular and mitochondrial preservation, a process synchronized by lipophilic nutritional antioxidants. As demonstrated by Pinilla-González et al. [33] through molecular dynamics simulations and cellular models, these lipid-soluble compounds preserve the physicochemical integrity, structural packing, and fluidic kinetics of endoplasmic reticulum (ER) membranes. By scavenging reactive oxygen species (ROS) and inhibiting lipid peroxidation within the ER lipid bilayer, these lipophilic antioxidants protect newly synthesized peptide chains and nascent oligomers from alternative oxidative modifications. This microenvironmental stabilization prevents subsequent proteotoxic degradation within the secretory pathway, thereby optimizing the folding efficiency and quaternary assembly of complex, multimeric pattern-recognition proteins.

When these fat-soluble coregulatory signaling pathways are restricted, host sensor competence fails. Under conditions of dietary vitamin A deficiency (VAD), the loss of retinoid-mediated transcription suppresses innate receptor expression and compromises mucosal barrier defenses. As documented by Amimo et al. [9], this environmentally induced immune restriction triggers mucosal epithelial atrophy and impairs dendritic cell homing, directly compromising local tissue barriers. When such environmental depletion coexists with host genetic variations, latent vulnerabilities are substantially exacerbated. This phenomenon is exemplified by Moumad et al. [34], whose genetic profiling demonstrated that under micronutrient scarcity, individual polymorphisms within innate immune sensor loci—specifically the C-type lectin receptor DC-SIGN (*CD209*)—elevate host susceptibility to severe mucosal infections and viral-mediated oncogenesis.

Vitamin D-driven transcriptional networks and lipophilic antioxidants jointly protect the folding and secretion of pattern-recognition molecules, and their loss compounds mucosal vulnerability. We next consider how the resulting variability in MBL synthesis is further shaped by inherited *MBL2* genotype.

### 3.3. Genetic Architecture, MBL2 Polymorphisms, and Epidemiological Penetrance

The genetic architecture regulating baseline systemic patterns of innate recognition molecules establishes the template upon which these fat-soluble micronutrient receptor axes operate. Quantitative mapping and multiplex ELISA screening in healthy reference cohorts confirm that individual variations in circulating mannan-binding lectin concentrations span up to a thousand-fold range, a heterogeneity strictly dictated by structural and promoter *MBL2* genotypes. As characterized by Troldborg et al. [5], single nucleotide polymorphisms within the promoter region (alleles H/L, X/Y) and exon 1 structural variants (alleles B, C, D, collectively termed O) alter the basic transcriptional rate and structural stability of the MBL peptide chain. This leads to functional deficiency in individuals carrying homozygous or compound heterozygous mutant genotypes.

Under chronic metabolic strain, this inherited heterogeneity influences clinical trajectories and tissue survival. Specific high-expressing *MBL2* polymorphisms, which maintain elevated fluid-phase lectin pathway activity, correlate with progressive renal deterioration, diabetic nephropathy, and microvascular complications in diabetic cohorts, reflecting how baseline genetic licensing shapes tissue responses to homeostatic disruption. This pathophysiological paradigm is robustly supported by a recent comprehensive meta-analysis by Sedghi et al. [35] and longitudinal data from Dørflinger et al. [36], which establish elevated circulating MBL levels as a major risk factor for microvascular disease and mortality in type 2 diabetes. Conversely, while individuals carrying low-expressing *MBL2* structural variants may experience lower complement-mediated vascular strain, they remain vulnerable to severe infectious complications when metabolic stress compromises alternative barrier systems.

The physiological relevance of this retinoid-dependent control is validated by clinical data demonstrating a functional gene–environment synergy within human cohorts. Kuhn et al. [15] conducted a randomized, double-blind, placebo-controlled trial investigating the interaction between structural *MBL2* polymorphisms and vitamin A status in infants exposed to vertical HIV transmission. In the placebo cohort, infants inheriting structural *MBL2* variants that compromise serum MBL concentrations and alter protein secretion exhibited an increased risk of vertical HIV infection (Odds Ratio [OR] = 3.09; 95% CI: 1.21, 7.86). Importantly, maternal vitamin A supplementation during the gestational and postpartum periods neutralized this genetically determined susceptibility, and removed the statistical association between low-expressing *MBL2* polymorphism status and transmission rates. This finding indicates a micronutrient-driven modulation of an inherited genetic defect in the innate immune machinery.

*MBL2* genotype sets the baseline range of circulating MBL, but its clinical consequences differ by context, raising vascular risk in diabetes while lowering it in some infectious settings—a context-dependence that the nutrigenetic framework below attempts to reconcile.

### 3.4. The Nutrigenetic Rescue Framework: Retinoid–Vitamin D Transcriptional Gating and Innate Immunocompetence

Integrating these molecular, transcriptomic, and clinical outcomes, we propose an explanatory framework governed by a dual-receptor transcriptional licensing mechanism, wherein the vitamin A-D receptor axis serves as a metabolic checkpoint regulating the biosynthesis, homeostatic competence, and pathogen-recognition efficiency of the mannan-binding lectin (MBL) interactome. Within this conceptual framework, the simultaneous engagement of RAR-RXR and VDR-RXR heterodimers within the nucleus is hypothesized to provide a necessary transcriptional license that sustains both the expression of the endoplasmic reticulum (ER) folding machinery and the integrity of mucosal epithelial barriers. Nascent MBL requires robust chaperone assistance to overcome structural vulnerability during early oligomerization. As a result, the functional capacity of the host to deploy this pattern recognition molecule against proteotoxic and infectious challenges is gated by retinoid and vitamin D receptor signaling thresholds.

At the intracellular level, concurrent nutritional deficits in vitamins A and D restrict the baseline expression of the helper chaperone network within the ER lumen. A compromised VDR-RAR status prevents the host from mounting an optimal, adaptive Unfolded Protein Response (UPR). This failure involves a cellular network modulated by retinoid signaling pathways, consistent with baseline receptor responses and chaperone adaptations described in broader models of retinoic acid exposure [31], while simultaneously precipitating the tissue-specific transcriptional dampening observed under severe micronutrient deficiency [8,9]. This restricted nutrigenetic status impairs the transcription of essential ER-resident chaperones, such as GRP78/BiP and protein disulfide isomerase (PDI). Consequently, nascent MBL polypeptide chains carrying structural variants are destabilized during early assembly. This structural compromise disrupts local divalent cation coordination within the secretory pathway, leaving these pattern recognition molecules highly vulnerable to misfolding and accelerated proteolysis, thereby depleting functional serum MBL levels.

Furthermore, this combined micronutrient restriction impairs the lipophilic antioxidant microenvironment required for secretory stability [32,33]. The subsequent accumulation of reactive oxygen species (ROS) within the ER bilayer subjects the destabilized, chaperone-depleted MBL chains to aberrant oxidative modifications. This multi-level structural disruption is hypothesized to lower the thermodynamic threshold for competitive zinc substitution within the evolutionarily conserved coordination spheres of MBL. This cation switch could theoretically neutralize the functional competence of the lectin pathway, converting an adaptive metabolic monitoring system into a state of accelerated host susceptibility to viral invasion or tissue failure under metabolic strain [34].

This proposed framework offers one plausible mechanistic interpretation of the notable gene–environment interaction documented by Kuhn et al. [15]. In their clinical evaluation of vertical HIV-1 transmission, Kuhn and colleagues demonstrated that infants carrying single-nucleotide polymorphisms in exon 1 of the *MBL2* gene (specifically the Arg52Cys, Gly54Asp, and Gly57Glu substitutions—collectively designated as allele *O*) exhibited a three-fold increase in susceptibility to mother-to-child HIV transmission within the placebo arm (OR: 3.09; 95% CI: 1.21, 7.86). This structural variant exerts a dominant-negative effect, disrupting the assembly of the collagen-like domain and rendering the protein highly vulnerable to metalloproteinase degradation, which reduces functional serum MBL concentrations by 5- to 10-fold in heterozygotes (A/O) and to undetectable levels in homozygotes (O/O). Importantly, exogenous maternal and perinatal administration of vitamin A and β-carotene completely neutralized this genetic risk (OR: 0.37; 95% CI: 0.15, 0.91), demonstrating a significant nutrigenetic rescue.

We propose that high-dose retinoid signaling operates as an exogenous compensatory mechanism capable of bypassing the inherited structural constraints of the *MBL2* variant allele *O*. In the immature neonatal immune system, where adaptive humoral responses are underdeveloped, the heavily glycosylated gp120 envelope of HIV presents a key target for MBL-mediated innate opsonization. Under vitamin A deficiency, the “two-hit” combination of maternal immune impairment and inherited host MBL depletion compromises frontline defense. Retinoid supplementation rescues this phenotype by driving the transcription of the ER helper machinery via robust RAR activation, optimizing the local folding microenvironment to favor the secretion of functional, high-molecular-weight MBL oligomers despite polymorphic constraints. Concurrently, vitamin A restores mucosal epithelial barrier integrity and mitigates the negative impacts of the acute-phase response on retinol-binding protein transport. This nutritional intervention selectively buffers structurally vulnerable polymorphic hosts against viral entry. This illustrates how targeted micronutrient synergy can alter the clinical penetrance of low-expressing immunogenetic genotypes and mitigate microvascular or infectious damage in high-risk populations [5,35,36].

Section 2 and Section 3 addressed two upstream determinants of lectin pathway competence—cation-dependent structural assembly and micronutrient-driven transcriptional licensing—both of which set a relatively stable, baseline capacity for the pathway. Circulating lectin pathway components do not, however, remain at a fixed baseline throughout life: their hepatic output and activation state are also under dynamic endocrine and metabolic control. The present section therefore shifts from these structural and licensing determinants to the pathway’s behavior as a dynamic sensor of metabolic and endocrine state, focusing on gestational and diabetic contexts in which this responsiveness has the clearest clinical relevance.

Vitamin A supplementation can clinically offset the infectious risk conferred by low-expressing *MBL2* genotypes, providing the clearest existing evidence for a gene-nutrient interaction in this pathway.

## 4. The Lectin Pathway as a Metabolic Sentinel: Gestational Diabetes, Autoimmunity, and Somatotropic Axis (GH/IGF-1) Regulation

### 4.1. Endocrine Regulation of Hepatic Synthesis via the Somatotropic and Thyroid Axes

The systemic availability of lectin pathway (LP) components is dynamically modulated by primary endocrine signaling networks rather than representing a static manifestation of host genetic architecture. The baseline hepatocyte synthesis of mannose-binding lectin (MBL) is directly regulated by neuroendocrine inputs. In vitro expression models utilizing the human HuH-7 hepatoma cell line established by Sørensen et al. [7] demonstrated that exposure to recombinant human growth hormone (rhGH), but notably not recombinant human insulin-like growth factor 1 (rhIGF-1), significantly stimulates MBL secretion into culture supernatants. Quantitative RT-PCR analyses confirmed that rhGH drives a threefold upregulation of specific *MBL2* mRNA transcription relative to *B2M* reference expression. This endocrine induction operates through pathways distinct from the classical, interleukin-6 (IL-6)-dependent acute-phase response, which yields only a marginal effect on MBL production in vitro, indicating a unique hormone-driven regulatory program governing pattern recognition molecule (PRM) production in the liver.

During gestational adaptation, maternal metabolic and hormonal shifts alter the secretory profile of these liver-derived PRMs. Deviations in circulating hepatokines during early gestation precede the clinical manifestation of metabolic and hepatobiliary dysfunction. In a meta-analysis encompassing 31 observational studies with over 4700 participants, Cai et al. [16] established that serum concentrations of ficolin-3 are significantly elevated in women during the second and third trimesters who subsequently develop gestational diabetes mellitus (GDM) compared to healthy pregnant controls (WMD 1.43 for the second trimester). This elevation correlates with maternal insulin resistance trajectories.

The clinical predictive value of this lectin pathway component during early gestational reprogramming is increased when integrated with adipocyte-derived signaling. In a prospective cohort study sampling maternal serum at 16–18 weeks of gestation, Yuan et al. [17] demonstrated that the ficolin-3 to adiponectin ratio serves as a biomarker with high sensitivity and specificity in this cohort for early homeostatic disruption. Utilizing a cut-off value of 1.06, the ficolin-3/adiponectin ratio achieved high diagnostic accuracy in predicting subsequent GDM development, yielding a sensitivity of 90.9% and a specificity of 96.5% (AUC 0.968, 95% CI: 0.940–0.995).

Beyond free circulating proteins and glucose intolerance, the gestational response enlists vesicular transport mechanisms during metabolic and hepatic complications. Data-independent acquisition (DIA) proteomics of serum-derived exosomes confirms that ficolin-3 is preferentially enriched within extracellular vesicles during physiological stress, such as intrahepatic cholestasis of pregnancy (ICP). In this specific pathology of gestational hepatobiliary disruption, exosomal ficolin-3 demonstrated high diagnostic promise, with receiver operating characteristic (ROC) curves achieving an area under the curve (AUC) value of approximately 0.90 [18]. Taken together, these insights underscore the role of the lectin pathway as an endocrine-responsive metabolic sentinel during the metabolic challenges of pregnancy.

Growth hormone, rather than IGF-1 or the classical acute-phase response, appears to be the principal endocrine driver of hepatic MBL synthesis, with ficolin-3 emerging as a candidate biomarker of gestational metabolic stress. We next examine how this metabolic sentinel function is altered in autoimmune and insulin-resistant states.

### 4.2. Immune-Metabolic Synergy in Type 1 Diabetes Autoimmunity

Beyond gestational metabolic disruption, the clinical utility of the lectin pathway as an active sentinel is evidenced by its interface with the autoimmune cascade driving pancreatic beta-cell destruction. The preclinical phase of Type 1 Diabetes (T1D) is characterized by the chronological emergence of specific islet autoantibodies. In a prospective pregnancy-birth cohort study tracking 1473 children with a first-degree relative with T1D, Couper et al. [37] demonstrated that persistent insulin autoantibodies (IAA), glutamic acid decarboxylase antibodies (GADA), and zinc transporter 8 autoantibodies (ZnT8A) follow distinct ontogenetic trajectories. Based on prospective monitoring of 1277 of these children, ZnT8A appeared as the initial autoimmune marker, either alone or in combination, in 43 (32%) of the 134 children who developed persistent islet autoantibodies. Importantly, as shown by Couper et al. [37], the progression from single seroconversion to clinical T1D depends heavily on the structural profile and binding characteristics of these biomarkers; persistent single ZnT8A restricted to standard ELISA detection formats usually appear after 4 years of age. Such cases signify a lower risk of rapid clinical progression compared to those appearing in multiple assay formats in younger children.

As this autoimmune process compromises glycemic stability, it shifts the systemic profile of the lectin pathway proteome. In the study by Jenny et al. [38], it was demonstrated that circulating concentrations of the initiating proteases, mannan-binding lectin-associated serine protease-1 (MASP-1) and MASP-2, are significantly elevated in both pediatric and adult individuals with established T1D compared to healthy controls. These proteases correlate directly with long-term glycemic control indexed by glycated hemoglobin (HbA1c) levels. Notably, the longitudinal volatility of these complement components reflects metabolic status: a marked intra-individual reduction in MASP-1 and MASP-3 levels is achieved when glycaemic control is substantially optimized, specifically when HbA1c is reduced by at least 10% from baseline [38]. Because MASP-1 and MASP-2 exhibit thrombin-like activity and directly engage with coagulation factors, their systemic elevation provides a direct link between chronic hyperglycemia and the prothrombotic state characteristic of T1D vascular complications.

This pathophysiological link between lectin pathway activation and metabolic status is further exacerbated by comorbid metabolic traits. In individuals with T1D, the presence of concomitant insulin resistance (IR), frequently termed the “double diabetes” phenotype, amplifies the underlying complement burden independent of HbA1c mediation. As reported by Kietsiriroje et al. [39], when stratified by the estimated glucose disposal rate (eGDR), a validated surrogate for insulin sensitivity, plasma levels of MASP-1, MASP-2, MASP-3, and their regulatory protein, mannose-binding lectin-associated protein (MAp44), increase in a strict stepwise fashion across worsening eGDR thresholds, and reach their highest concentrations in patients with greater degrees of IR. Furthermore, therapeutic or behavioral interventions investigated in Kietsiriroje’s study that successfully improve insulin sensitivity (increasing eGDR) over a 6-month period yield significant reductions in the active proteases MASP-1, MASP-2, and MASP-3. Interestingly, plasma levels of the regulatory protein MAp44 remain unaffected by improvements in insulin sensitivity, pointing to a rigid, structural alteration in complement regulation during chronic insulin-resistant states in T1D.

MASP-1/2 levels track glycemic control in type 1 diabetes and rise further with insulin resistance, linking innate complement activity to metabolic as well as autoimmune status. The next section asks whether this activation is driven mainly by inherited genotype or by hyperglycemia itself.

### 4.3. Genetic Susceptibility and Independent Local Activation Mechanisms in Diabetic Microvasculopathy

The transition from subclinical metabolic strain to established microvascular endpoints highlights distinct genetic and functional architectures separating Type 1 (T1D) and Type 2 Diabetes (T2D). Epidemiological synthesis confirms that systemic lectin pathway activation scales with the severity of secondary tissue damage. In a meta-analysis of 28 observational studies, Sedghi et al. [35] established that elevated circulating MBL strongly associates with increased vascular complications (pooled Hazard Ratio [HR] = 1.44, 95%CI: 1.07–1.95) and all-cause mortality (pooled HR = 1.52, 95%CI: 1.07–2.16), with the most pronounced risk elevation observed for diabetic nephropathy (pooled HR = 2.16, 95%CI: 1.52–3.08). This proteolytic burden is tightly constrained by host genetics; in T2D cohorts followed prospectively for 12 years, individuals carrying high MBL-expressing genotypes exhibit faster renal deterioration, characterized by progressive declines in estimated glomerular filtration rate (eGFR) and accelerated plasma creatinine elevations (*p* = 0.029) [36,40,41]. This genetic hardwiring extends to the initiating complexes, where specific single nucleotide polymorphisms within the *MASP1* gene (rs874603, rs72549254, rs3774275, rs67143992, rs850312) modulate the circulating concentrations of MASP-1, MASP-3, and the inhibitory protein MAp44 [42].

Intriguingly, the induction of diabetes independently overrides these baseline genetic constraints. Hyperglycemia drives autonomous elevations of plasma MASP-1 (*p* = 0.003) [42], promoting local complement activation, leukocyte recruitment, and aberrant angiogenesis. This local metabolic override plays a documented role in the pathogenesis of diabetic retinopathy, where systemic MBL elevations serve as a distinct biomarker for advanced tissue damage [43]. At the terminal tissue interface, however, experimental gene-ablation models reveal a clear uncoupling between local histological protection and systemic complement activation. In a streptozotocin-induced murine model of advanced T1D, targeted deletion of the primary executing protease (*Masp2*^−/−^) curtails glomerular mesangial matrix expansion, reducing it to 21.1% of the glomerular area compared to 25.2% in wild-type controls (*p* = 0.001), and stabilizes plasma cystatin C kinetics by mitigating early diabetes-induced hyperfiltration [19]. Notably, this specific MASP-2 deficiency fails to rescue the renal cortex from diabetes-induced hypertrophy, macroalbuminuria, or the localized upregulation of profibrotic (*Tgfb1*) and oxidative stress (*Cybb*) transcripts. This phenotypic divergence indicates that while MASP-2 drives focal structural remodeling in the mesangium, parallel pathobiological pathways, independent of lectin-pathway-mediated C3 activation, continue to propagate diffuse diabetic kidney injury.

*MBL2* genotype and hyperglycemia act as separate, additive drivers of complement activation in diabetes, and animal data suggest MASP-2-independent pathways also contribute to renal injury.

### 4.4. The Somatotropic-Gestational Sentinel Hypothesis and Alternative Complement Bypasses

Integrating these endocrinological, gestational, and microvascular profiles, we propose an integrated framework wherein the lectin pathway functions as a metabolic sensor whose circulating competence is licensed by the somatotropic axis and dynamically altered by glucotoxic strain. Within this framework, physiological fluctuations in GH and IGF-1 availability are hypothesized to dictate baseline hepatic *MBL2* transcription. Paralleling this, peripheral growth hormone resistance or altered IGF-1 bioavailability, characteristic of advanced metabolic syndromes, would presumably suppress this baseline transcriptional licensing. This endocrine-driven suppression could impair the availability of circulating pattern recognition complexes, altering host surveillance capacity prior to the onset of overt inflammatory complications.

During gestational adaptation, maternal hyperglycemia induces profound trophoblast and endothelial stress. The systemic elevation of circulating ficolin-3 [16] and its prominent sorting into serum-derived exosomes during gestational metabolic stress [18] may represent an orchestrated compensatory response. We hypothesize that a concurrent, fat-soluble vitamin deficit during this developmentally sensitive window impairs the endoplasmic reticulum (ER) chaperone machinery required to process this increased ficolin-3 workload, thereby accelerating protein misfolding within the secretory pathway and exacerbating local cellular stress.

Furthermore, we hypothesize that the upregulation of circulating MASPs in T1D and T2D represents an accelerated proteolytic feedback loop driven by systemic glucotoxicity and advanced glycation end-product (AGE) accumulation. While the initial triggering of islet autoantibodies, including the early-onset ZnT8A seroconversion patterns monitored by Couper et al. [37], remains rooted in adaptive autoimmunity, the downstream systemic vasculopathy appears to involve an innate immune amplification step. Sustained metabolic stress, characterized by elevated HbA1c and concurrent insulin resistance, could theoretically alter the glycosylation patterns of circulating PRMs, lowering the thermodynamic threshold for autonomous, autokinetically driven MASP activation.

This framework provides a plausible mechanistic explanation for the puzzling persistence of tubulointerstitial fibrosis and macroalbuminuria observed in *Masp2*^−/−^ knockout models under diabetic strain [19]. We propose that the progression of diabetic microvascular complications relies on autonomous, tissue-specific complement bypass mechanisms rather than a single, linear proteolytic cascade. Chronic systemic glucotoxicity and AGE accumulation might trigger direct, MASP-2-independent activation of C3 by MASP-1, or alternatively initiate the properdin-mediated alternative pathway amplification loop directly on the altered endothelial surface. This architectural redundancy explains why high MBL-expressing genotypes consistently drive multi-decade renal and retinal deterioration [35,36], rendering downstream single-protease blocks therapeutically insufficient. A sustained deficiency in fat-soluble vitamins during this chronic metabolic shift would substantially impair the physiological synthesis or stability of endogenous complement regulators, such as MAp44 or CD59. This deficit would deprive the microvascular endothelium of its protective shield, converting a genetically determined high-activation lectin pathway profile into an uncontrolled driver of permanent tissue ischemia and accelerated microvascular failure.

Section 4 showed that metabolic and endocrine shifts alter lectin pathway activity in diabetes and pregnancy, establishing the pathway as a metabolic sensor at the level of the individual patient. The final section broadens this perspective from individual metabolic physiology to public health: because the same activation mechanisms that respond to metabolic stress are also engaged by acute viral infection, and because both processes converge on measurable circulating biomarkers, we now connect these metabolic profiles to lectin pathway hyperactivation in infection and to the diagnostic technologies that could translate these biomarkers into clinical and public health practice.

This endocrine- and glucotoxicity-driven synthesis remains a framework proposed by the authors to explain existing observations, not a demonstrated mechanism.

## 5. Public Health and Diagnostic Innovation: Epidemiological Evidence and Rapid Nanoplasmonic Detection

### 5.1. Endothelial Dysfunction, Angiopathy, and Host Genetic Architecture

The systemic availability of lectin pathway (LP) components directly modulates the structural integrity and barrier capacity of the vascular endothelium. Mannan-binding lectin-associated serine protease-1 (MASP-1), the most abundant enzymatic component of this cascade, regulates endothelial barrier functions independently of intravascular antimicrobial defense. In vitro modeling utilizing real-time micro-electric sensing demonstrates that exposure to active MASP-1 induces a dose-dependent decrease in human umbilical vein endothelial cell (HUVEC) monolayer impedance [44]. This reduction in transendothelial electrical resistance (TEER) accelerates paracellular macromolecular transport, confirming a direct increase in endothelial permeability. This barrier disruption is initiated by the proteolytic cleavage of the *N*-terminal exodomain of protease-activated receptor 2 (PAR-2). The resulting tethered ligand triggers Gq-protein-coupled intracellular calcium (Ca^2+^) mobilization, protein kinase C (PKC) activation, and the transcriptional translocation of nuclear factor kappa B (NF-κB) mediated by the p38 mitogen-activated protein kinase (MAPK) pathway [44].

This permeabilizing effect is amplified when the endothelium is subjected to concurrent localized ischemic stress. Under tissue hypoxia, a defining feature of advanced atherosclerotic lesions, ischemic stroke, and acute myocardial infarction (AMI), the vascular endothelium undergoes metabolic and structural phenotype switching [45]. Hypoxia independently suppresses the migratory and wound-healing capacities of endothelial cells while altering adhesion molecule topography through the transcriptional upregulation of intercellular adhesion molecule-1 (ICAM-1) and the downregulation of ICAM-2. While hypoxia and MASP-1 operate via distinct biochemical pathways to accelerate paracellular leakage and stimulate the release of chemokine ligands such as interleukin-8 (IL-8/CXCL8) and growth-regulated oncogene-alpha (GRO-α/CXCL1), they manifest a pathophysiological synergy regarding structural vascular remodeling [45]. The convergence of hypoxia and MASP-1 disrupts capillary-like vascular network integrity and drives the surface expression of E-selectin, a key adhesion molecule regulating neutrophil homing and rolling. This cooperative amplification is driven by an upstream, hypoxia-induced upregulation of PAR-2 transcripts, providing an expanded substrate for MASP-1-mediated receptor cleavage and subsequent signaling co-activation [45]. Consequently, complement hyperactivation within anoxic vascular beds accelerates atherosclerotic plaque vulnerability and expands ischemic injury zones.

The clinical relevance of these protease-driven endothelial perturbations is supported by plasma profiling in clinical cohorts. Systematic quantification of circulating lectin pathway components shows that altered balances of MASPs and their endogenous regulatory proteins correlate with macrovascular and cerebrovascular endpoints [3]. Significant shifts in plasma MASP-1 and MASP-2 levels occur in patients with established coronary artery disease (CAD), acute myocardial infarction (MI), and acute ischemic stroke. Concurrently, plasma MASP-1 levels peak in subacute MI patients but drop to their lowest values in acute stroke cohorts, whereas MASP-2 levels decrease in both MI and stroke compared to healthy controls [3]. Because MASP-1 and MASP-2 are positioned at the intersection of inflammatory signal transduction and fibrin clot formation, their systemic bioavailability and relative complexing with C1-inhibitor (C1-INH) modulate individual thromboinflammatory thresholds, dictating the ultimate severity of myocardial and cerebral tissue ischemia.

Delineating these epidemiological patterns requires a precise mapping of the underlying human genetic architecture. While environmental, metabolic, and hypoxic stressors dictate the rate of complement activation, baseline genetic diversity establishes the physiological boundaries of this response. Hereditary deficiencies and structural polymorphisms within complement-activating genes underscore the developmental and immunophysiological consequences of altered innate immunity [46]. Beyond standard vulnerability to opportunistic microorganisms or autoimmune conditions, specific mutations within the *MASP1* gene, which codes for MASP-1, MASP-3, and the regulatory protein MAp44 via alternative splicing, are linked to complex autosomal-recessive developmental anomalies, such as the 3MC (Carnevale, Mingarelli, Malpuech, and Michels) syndrome [46,47]. This biological penetrance demonstrates that genetically determined aberrations in lectin-associated serine proteases, particularly the loss of MASP-3-mediated activation of alternative pathway factor D, destabilize homeostatic networks outside of pathogen defense.

This genetic vulnerability is further illustrated by primary immunodeficiencies embedded within the population, such as functional MASP-2 deficiency [13]. In Caucasian populations, this autosomal recessive trait is driven by homozygosity for the *MASP2* c.359A>G missense mutation (p.D120G; rs72553870), which occurs with an estimated frequency of approximately 6 per 10,000 individuals. The biochemical phenotype of a case involving a Polish female homozygous for the D120G allele underscores the structural nuances of this deficiency [13]. Although structural MASP-2 protein remains detectable at low baseline concentrations, the substitution of a glycine residue for aspartic acid at position 120 destabilizes the calcium-dependent epidermal growth factor (EGF)-like domain. Consequently, the mutated protease is incapable of associating with its upstream pattern recognition molecules, mannan-binding lectin (MBL) and ficolins, resulting in a complete abolition of MBL-dependent complement activation.

The existence of single-nucleotide-driven phenotypes and multi-protein coding defects within the human population demonstrates that individual inflammatory reactivity is genetically determined. This genetic architecture is further modulated by broader systemic gene interaction networks. On a macro-genomic scale, machine learning analyses and multi-cohort bioinformatic evaluations have identified key hub genes, such as those regulating M1 macrophage polarization (*LAPTM5*, *CSF1R*, *C1QC*) or altering cellular pharmacokinetic pathways (*SLCO2B1*), that dictate tissue infiltration patterns and modify local immune responses [20,48,49]. When these variable genomic backgrounds encounter acute metabolic or hypoxic triggers, they yield divergent clinical outcomes. The heterogeneity observed between individuals suffering from angiopathic complications limits the utility of aggregate diagnostic paradigms. Consequently, public health frameworks require high-throughput, point-of-care screening technologies capable of mapping this diverse genetic and proteomic landscape before irreversible macrovascular failure occurs.

MASP-1 directly compromises endothelial barrier function, an effect amplified by hypoxia, against a background of rare but clinically important complement gene mutations. We next consider how this same proteolytic machinery is co-opted during acute viral infection.

### 5.2. Viral-Induced Lectin Pathway Hyperactivation and Thromboinflammatory Pathophysiology

Acute viral infections, particularly those driven by severe acute respiratory syndrome coronavirus 2 (SARS-CoV-2), act as potent systemic triggers for complement lectin pathway hyperactivation. Molecular binding assays demonstrate that structural proteins of SARS-CoV-2 interact directly with host pattern recognition molecules to initiate cascade activation independently of adaptive immunity [21]. The viral spike (S) protein, specifically its receptor-binding domain (RBD), and the nucleocapsid (N) protein serve as ligands for MBL, ficolin-2, and collectin-11 [21]. Upon glycan-mediated recognition or direct protein-protein binding, these viral complexes induce conformational changes in MBL-associated serine proteases, promoting the autoactivation of MASP-1 and subsequent cleavage-mediated activation of MASP-2 [21,22]. This physical interaction is amplified by the viral N protein, which binds directly to the effector enzyme MASP-2, establishing an autonomous platform for downstream C4 and C3 deposition within affected tissues [21].

The biochemical consequences of this pathogen-driven initiation extend beyond innate immune defense, directly driving microvascular endothelial injury and systemic thromboinflammation. Once activated by viral antigens, MASP-1 and MASP-2 operate at the crossroad of the complement and coagulation cascades [22]. MASP-1 exhibits a substrate specificity that allows it to cleave fibrinogen and activate prothrombin, simulating the enzymatic activity of thrombin and accelerating intravascular fibrin deposition, while concurrently cleaving protease-activated receptors (PARs) to drive microthrombogenesis. In parallel, the viral N protein acts as a pathogen-associated molecular pattern (PAMP) that binds to Toll-like receptor 2 (TLR2), activating the NF-κB and MAPK signaling pathways [23]. This interaction significantly upregulates the expression of soluble intercellular adhesion molecule-1 (sICAM-1) and vascular cell adhesion molecule-1 (sVCAM-1), compounding MASP-mediated endothelial cell dysfunction and promoting a prothrombotic state within the microvascular networks [23]. This cooperative interaction between viral components and host serine proteases shifts the vascular equilibrium toward intravascular microthrombogenesis, contributing to the clinical manifestations of advanced angiopathy and tissue ischemia observed during severe viral syndromes.

Plasma profiling and longitudinal cohort monitoring confirm the clinical relevance of these biochemical pathways in patients with acute viral infections. Systematic quantification of circulating lectin pathway components shows that severe clinical phenotypes correlate with distinct proteomic shifts and complement consumption patterns. In prospectively followed COVID-19 cohorts, plasma concentrations of MASP-2 significantly increase during the acute phase of infection, correlating directly with the terminal complement complex (TCC), ficolin-2, ficolin-3, and C-reactive protein (CRP) [50]. This systemic elevation reflects an acute-phase response to viral-mediated tissue damage [50]. Dynamic modeling indicates that complement hyperactivation occurs early (7–14 days post-symptom onset) and is intensely mediated by a synergy between the lectin and alternative pathways, rather than the classical pathway, where alternative pathway factor Bb correlates directly with thromboinflammation and severe clinical outcomes [51].

When these acute viral triggers encounter compromised host end-organ systems, the pathophysiological outcomes are further modulated by underlying medical conditions. In cohorts with preexisting chronic kidney disease, plasma levels of lectin pathway proteins show significant alterations that correlate with COVID-19 severity, heavily influenced by host genetic and ethnic backgrounds [52]. However, the exact pathogenetic weight of the MBL-mediated lectin pathway remains a subject of nuance; while markers of lectin pathway activation, such as the MASP-1/C1-INH complex and C4d, correlate with acute disease severity, longitudinal evaluations indicate that genetic variations within the *MBL2* gene do not show a consistent, clinically meaningful association with overall mortality or the development of long COVID [53]. This suggests that while the lectin pathway is systematically activated during acute infection, its individual genetic architecture may play a secondary role relative to the generalized thromboinflammatory response driven by alternative pathway amplification and direct viral PAMP-mediated endothelial damage.

Bioinformatic network analyses have mapped the overlapping transcriptomic profiles of severe viral infections and major comorbidities, identifying overlapping hub genes [12]. Protein–protein interaction networks matching COVID-19 with hypertension, diabetes mellitus, and coronary artery disease have identified 11 key shared genetic markers, including *MBL2*, *TLR4*, *NLRP3*, *IL6*, and *AGT*, with *IL6* and *AGT* functioning as the central nodes regulating the host immune response and cytokine activity [12]. These shared genetic profiles modulate host inflammatory reactivity and predispose specific subpopulations to severe endothelial failure when exposed to acute viral stressors. The biological variability observed in individual thromboinflammatory thresholds highlights the necessity for high-throughput diagnostic screening to map individual proteomic and genetic profiles, enabling personalized therapeutic interventions before irreversible vascular and organ failure occurs [12,53].

SARS-CoV-2 proteins directly engage lectin pathway components to drive thromboinflammation, although *MBL2* genotype itself appears to have limited independent effect on COVID-19 outcomes. This gap between population-level genetic risk and individual clinical course motivates the diagnostic approach considered next.

### 5.3. Nanoplasmonic Biosensors as Point-of-Care Diagnostic Innovations

Translating complement lectin pathway (CLP) assessment into routine clinical practice and epidemiological surveillance requires a shift from conventional, centralized methodologies. Standard techniques for quantifying CLP components, primarily enzyme-linked immunosorbent assays (ELISA) and functional hemolytic assays, are constrained by prolonged turnaround times, high labor intensity, and the necessity of specialized laboratory infrastructure. These limitations preclude their deployment in high-throughput population screenings, emergency department triage, or resource-limited settings where immediate risk stratification is vital.

To address these analytical bottlenecks, microfluidic and bioengineering platforms increasingly utilize localized surface plasmon resonance (LSPR) biosensors designed for point-of-care (POC) diagnostics. These nanoplasmonic detection systems enable real-time, label-free quantification of mannan-binding lectin (MBL), ficolins, and MBL-associated serine proteases (MASPs) from microliter-scale clinical samples. The biosensor architecture typically relies on nanostructured metallic substrates (such as gold nanodisks) functionalized with specific carbohydrate ligands or monoclonal antibodies. Molecular binding events alter the local refractive index at the immediate dielectric interface of the nanostructure, inducing a quantifiable red-shift in the LSPR peak, which yields precise proteomic readouts within minutes.

From a clinical and public health perspective, rapid proteomic profiling facilitates the immediate identification of functional immunodeficiencies and genetic variants. The diagnostic complexity of this pathway is heightened by the presence of multiple splice variants and non-enzymatic proteins sharing high sequence homology, such as MAp19, MAp44, and MASP-3, which act as crucial regulators of the pathway [46]. POC devices can rapidly screen for individuals with primary homozygous *MBL2* or *MASP2* mutations (such as the *MASP2* D120G variant), which lead to a near-complete loss of functional lectin pathway activity [4,13]. While these deficiencies often present with low clinical penetrance in healthy individuals, they are considered vital disease modifiers that compromise innate immunity during concurrent metabolic stress, immunosuppression, or systemic infectious challenges [13,46]. In such high-risk scenarios, high-throughput nanoplasmonic assays can monitor real-time complement consumption and acute proteomic shifts, providing essential biochemical data before clinical deterioration occurs. Integrating these microfluidic assays into clinical workflows transitions the evaluation of the lectin pathway from a specialized research tool into a predictive biomarker panel for systemic homeostasis.

LSPR biosensing remains a promising but still preclinical concept for translating lectin pathway biomarkers into rapid point-of-care diagnostics.

## 6. Conclusions and Future Perspectives

The lectin pathway (LP) of complement activation represents a crucial biochemical axis integrating innate immunity, nutritional regulation, and metabolic endocrinology. This pathway functions as a systemic metabolic sensor that is directly modulated by micro- and macronutrients. Divalent cations exert stringent conformational control over LP activation: physiological concentrations of calcium (Ca^2+^) are required for structural stability and pattern recognition, whereas zinc (Zn^2+^) is proposed to exert a concentration-dependent inhibitory effect on protease homodimerization and catalytic activity, a mechanism extrapolated from studies of other serine proteases and not yet directly demonstrated for lectin pathway proteases. Transcriptional control of these immune components is regulated via the retinoid–vitamin D receptor axis, where calcitriol and retinoic acid signaling pathways demonstrate nutrigenetic synergy via RXR/VDR heterodimers to maintain epithelial and mucosal barrier competence.

In metabolic pathophysiology, aberrant or sustained LP activation correlates with the progression of diabetes mellitus and chronic gestational hyperglycemia. Chronic subclinical inflammation and persistent glycemic fluctuations accelerate endothelial dysfunction, driving microvascular complications including diabetic retinopathy and nephropathy. The severity of these clinical phenotypes is mediated by distinct serine protease profiles and individual genetic susceptibility [35,36,43]. During gestational metabolic derangements, compensatory molecular mechanisms intersect with these cascades; specifically, upregulated galectin-3/FOXC1 signaling modulates complement-related pathways and mitigates high-glucose-induced trophoblast apoptosis to preserve placental integrity.

Translating these biochemical insights into clinical and epidemiological strategies requires addressing three core research priorities:

1. Quantification of complement consumption kinetics: Longitudinal studies must track the real-time consumption rates of functional cascade components during overlapping infectious and metabolic crises. Establishing precise biochemical thresholds is essential to differentiate protective, acute-phase activation from self-destructive thromboinflammatory pathology and profound hypocomplementemia [50,51].
2. Clinical validation of nutrigenetic interventions: Targeted clinical trials are required to evaluate personalized micronutrient strategies (specifically Vitamin A, Vitamin D, and zinc supplementation) tailored to individual host genotypes. While neonatal whole-blood zinc content shows no significant association with MBL levels in healthy newborns [26], targeted multi-therapeutic antioxidant and micronutrient synergy may counteract hyperactivation phenotypes [33], particularly in high-risk groups or individuals with specific primary immunodeficiencies such as homozygous MASP-2 deficiency [13]. No published RCT has yet used LP components (MBL, ficolins, MASPs) as a primary or secondary endpoint in diabetic or gestational-diabetic populations. The closest available evidence comes from independent meta-analyses of zinc supplementation and vitamin D supplementation on general inflammatory/oxidative-stress markers in adults and in pregnancy, respectively [54,55], neither of which reports LP-specific proteins or was conducted in a GDM-specific population, underscoring this as a priority gap for future interventional research.
3. Integration of point-of-care epidemiological screening: Rapid nanoplasmonic assays represent a potential future application for routine clinical surveillance that, pending further assay development and prospective clinical validation for lectin-pathway measurements, could aid early detection of metabolic drift, endothelial stress, and subclinical innate immune hyperactivation before the onset of irreversible macrovascular or microvascular disease.

In conclusion, this review bridges nutritional biochemistry with metabolic endocrinology and outlines diagnostic technologies—notably LSPR biosensing—that remain largely preclinical and require prospective clinical validation before implementation; once validated, they could support a shift from empiric management toward biomarker-driven, point-of-care stratification, helping to mitigate the progression of both infectious thromboinflammation and chronic metabolic complications.

## Figures and Tables

**Table 1:** Key lectin pathway components: structure, principal function, and associated clinical conditions.

Component	Structural Class	Principal Function	Associated Clinical Conditions
MBL	Collectin (collagen-like + CRD)	Ca^2+^-dependent carbohydrate recognition; opsonization; MASP docking	MBL deficiency (recurrent infection); diabetic nephro-/retinopathy (high-expressing genotypes)
Ficolin-1/-2/-3	Collagen-like + fibrinogen-like domain	GlcNAc/acetyl-group recognition; MASP docking	Gestational diabetes biomarker (ficolin-3); intrahepatic cholestasis of pregnancy
CL-11	Collectin	Pattern recognition, MASP docking	Developmental anomalies (3MC syndrome, via COLEC11)
MASP-1	Serine protease	Autoactivator of MASP-2; PAR cleavage; endothelial activation	Elevated in T1D/T2D, CAD, stroke; 3MC syndrome
MASP-2	Serine protease	Cleaves C4/C2; generates C3 convertase	MASP-2 deficiency (immunodeficiency); COVID-19 severity
MASP-3	Serine protease (MASP1 splice variant)	Activator of pro-factor D (alternative pathway)	3MC syndrome; modulated in T1D insulin resistance
MAp44/MAp19	Non-enzymatic splice variants	Endogenous competitive inhibitors of MASP activation	Elevated with insulin resistance in T1D

Abbreviations: 3MC: 3MC syndrome; C2: Complement component 2; C3: Complement component 3; C4: Complement component 4; CAD: Coronary artery disease; Ca^2+^: Calcium ion; CL-11: Collectin-11; *COLEC11*: Collectin subfamily member 11 gene; COVID-19: Coronavirus disease 2019; CRD: Carbohydrate recognition domain; GlcNAc: N-acetylglucosamine; MAp19: 19 kDa MBL-associated protein; MAp44: 44 kDa MBL-associated protein; MASP-1/2/3: MBL-associated serine protease (-1/-2/-3); *MASP1*: MBL-associated serine protease 1 gene; MBL: Mannose-binding lectin; PAR: Protease-activated receptor; T1D: Type 1 diabetes mellitus; T2D: Type 2 diabetes mellitus.

**Table 2:** Overview of the explanatory frameworks proposed in this review, and their current evidence status.

Conceptual Framework	Core Claim	Key Supporting Literature	Evidence Status
Dual-Cation Dichotomy Framework	Ca^2+^ is architecturally required for PRM-MASP assembly; Zn^2+^ is proposed as a catalytic/homeostatic rheostat	Ca^2+^ arm: [1,2,4,6]. Zn^2+^ arm: indirect precedent only [14]	Ca^2+^ arm: well established. Zn^2+^ arm: authors’ hypothesis, not yet tested directly on MASPs
Nutrigenetic Rescue Framework	Vitamin A/D receptor signaling transcriptionally licenses MBL/ficolin synthesis and ER chaperone capacity, buffering low-expressing *MBL2* genotypes	[8,9,10,11,15]	Partially supported: transcriptomic data are direct; the clinical gene–nutrient interaction rests on one RCT (Kuhn et al. [15])
Somatotropic-Gestational Sentinel Hypothesis	GH/IGF-1 axis licenses hepatic *MBL2* transcription; gestational ficolin-3 elevation is a compensatory endocrine-responsive sentinel	[7,16,17,18]	Hypothesis-generating synthesis; individual observations are direct, the integrative framework is the authors’ proposal
LSPR point-of-care diagnostic framework	Nanoplasmonic biosensors could enable rapid population-level LP screening	General LSPR literature; clinical rationale from [3,13,19,20,21,22,23]	Conceptual/preclinical: no clinical validation of LP-specific LSPR assays has been published

Abbreviations: Ca^2+^: Calcium ion; ER: Endoplasmic reticulum; GH: Growth hormone; IGF-1: Insulin-like growth factor 1; LP: Lectin pathway; LSPR: Localized surface plasmon resonance; MASP: MBL-associated serine protease; MBL: Mannose-binding lectin; *MBL2*: Mannose-binding lectin 2 gene; PRM: Pattern recognition molecule; RCT: Randomized controlled trial; Zn^2+^: Zinc ion.

## Data Availability

No new data were created or analyzed in this study. Data sharing is not applicable to this article.
